# Surgical strategy for metastatic spinal tumor patients with surgically challenging situation

**DOI:** 10.1097/MD.0000000000029560

**Published:** 2022-07-08

**Authors:** Hong Kyung Shin, Myeongjong Kim, Subum Lee, Jung Jae Lee, Danbi Park, Sang Ryong Jeon, Sung Woo Roh, Jin Hoon Park

**Affiliations:** a Department of Neurological Surgery, Asan Medical Center, University of Ulsan College of Medicine, Seoul, Republic of Korea; b Department of Neurosurgery, Seongnam Citizens Medical Center, Seongnam, Republic of Korea; c Department of Neurosurgery, Korea University Anam Hospital, Korea University College of Medicine, Seoul, Republic of Korea; d Department of Neurosurgery, Gangneung Asan Hospital, University of Ulsan College of Medicine, Gangneung, Republic of Korea.

**Keywords:** clinical outcome, metastatic spinal tumor, radiological outcome, surgical strategy, surgically challenging situation

## Abstract

The incidence of spinal metastasis is increasing as cancer patients live longer owing to the improvement of cancer treatments. However, traditional surgery (TS) which fixates at least 2 levels above and 2 levels below the affected vertebrae is sometimes difficult to perform as it is burdensome to the patients. In this article, we introduce our experience and strategy in treating spinal metastasis, focusing particularly on challenging cases.

We retrospectively reviewed the data of 110 patients who underwent spinal surgery for metastatic spinal tumors from April 2018 to March 2020. Among them, 5 patients who received anterior approach surgery were excluded. The remaining 105 patients were enrolled. In addition to TS, we also performed cervical pedicle screw, cervicothoracic junction fixation, thoracolumbar short fixation, and decompression surgery, depending on the characteristics of the tumor. The overall survival was analyzed, and the local tumor control rate was evaluated using magnetic resonance imaging. Perioperative clinical characteristics including Spine Oncology Study Group Outcomes Questionnaire, visual analog scale, Eastern Cooperative Oncology Group performance score, and Karnofsky Performance Score were also investigated.

The overall survival rate was 57.9% at 1 year, and the local tumor control rate was 81.1% after surgery. There was a statistically significant difference according to the type of the tumor in the survival analysis: the overall survival rates were 72.7% for favorable tumors and 48.6% for unfavorable tumors at 12 months after surgery (*P* = .04). Spine Oncology Study Group Outcomes Questionnaire, visual analog scale, Eastern Cooperative Oncology Group performance score, and Karnofsky Performance Score was improved after surgery. All surgical methods, including TS, cervical pedicle screw, cervicothoracic junction fixation, thoracolumbar short fixation, and decompression surgery, showed good clinical and radiological outcomes.

Optimized surgical methods show similarly good clinical outcomes in managing spinal metastasis as TS.

## 1. Introduction

The skeletal system is the third most common site of cancer metastasis, following the lungs and liver.^[1]^ Among the skeletal system, the spine is the most common site of bone metastasis.^[1,2]^ Recent advances in cancer treatment has allowed increased survival time for patients, resulting in more patients developing spinal metastasis.^[3,4]^ Recently, the treatment of spinal metastatic tumors, including surgery, radiotherapy (RT), stereotactic radiosurgery (SRS), and chemotherapy, has tremendously improved.^[5]^

The objective of traditional surgery (TS) is to excise the tumor with a radical margin with multiple level pedicle screw fixation. Such treatment results in high morbidity and complication rates, especially in patients with numerous tumor-associated comorbidities.^[6,7]^ In addition, it is difficult to decide on the appropriate surgical level in patients with multiple spinal metastasis. Patients with spinal metastases usually have poor general condition owing to treatment with concomitant chemotherapy, immunosuppression, malnutrition, and significant pain; considering this and the survival rate after spinal metastasis,^[8,9]^ palliative surgery could be a good treatment of choice, as it is less invasive and safe. In this context, there have been many efforts to perform less invasive surgeries, including laminectomy, separation surgery, vertebroplasty, decompression, and instrumentation without fusion,^[7,10–12]^ although the choice of treatment is still debatable.

Surgery of metastatic spinal tumors should be decided based on the neurologic function, subjective pain, quality of life, and life expectancy of each patient. In this article, we aimed to present the various surgical strategies to overcome such difficult situations, which cannot be controlled by TS in metastatic spinal tumors.

## 2. Materials and Methods

**Figure 1.**
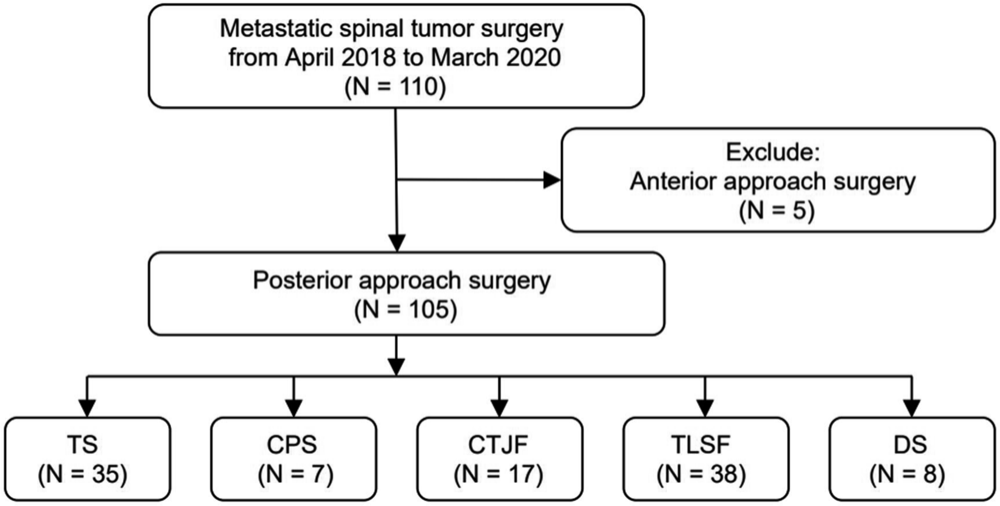
Flow chart showing patient selection. CPS = cervical pedicle screw, CTJF = cervicothoracic junction fixation, DS = decompression surgery, TLSF = thoracolumbar short fixation, TS = traditional surgery.

**Figure 2.**
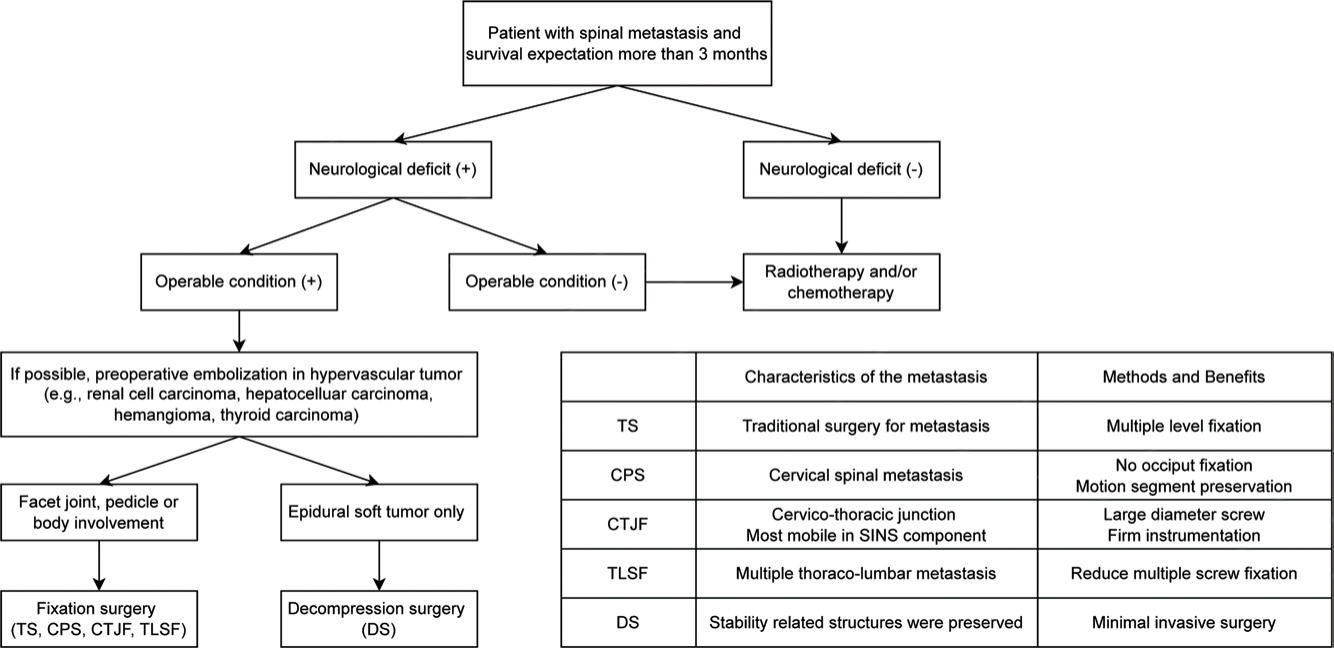
Schematic flow algorithm of decision-making in spinal metastasis.

We retrospectively reviewed the records of 110 patients with metastatic spinal tumors who underwent surgery in a single tertiary center from April 2018 to March 2020. The indications for surgery were mostly neurologic deficits with unbearable pain, and all patients were regarded as having tolerable status for surgery, with a life expectancy of >3 months considering their general health condition and status of primary cancer. Among them, 5 patients who received anterior approach surgery were excluded. The remaining 105 patients were enrolled in the study. The surgical protocol comprised TS, cervical pedicle screw (CPS), cervicothoracic junction fixation (CTJF), thoracolumbar short fixation (TLSF) and decompression surgery (DS) (Fig. 1). A schematic flow algorithm explaining the decision-making in our study is illustrated in Figure 2. This study was performed after institutional review board (IRB) approval, and the IRB waived the requirement of obtaining informed consent for this study.

TS was performed for 35 patients with solitary metastasis in good general condition. CPS was conducted in 7 patients with cervical metastasis who required firm instrumentation for instability. CTJF was performed in 17 patients. It is unique in its curve as the cervicothoracic junction is where the sagittal curve changes and solid instrumentation is mandatory: pedicle screw was preferred for instrumentation. TLSF was performed in the thoracolumbar spine, to lessen the fixation level. Since multiple spinal metastasis is not rare and long level surgery can cause undesirable complications, surgical level was reduced as much as possible. For the same reason, DS was also performed in 8 patients with small metastases without instability. The anterior column was supported posteriorly with a mesh cage after corpectomy, or an interbody cage after discectomy. More specifically, titanium mesh cages or polyetheretherketone cages with bone chips were used. To enhance bone fusion, a demineralized bone matrix (Allomix, CGBIO, Republic of Korea) was used.

Preoperative demographics and clinical data, including age, sex, primary tumor, offending level of the lesion, history of neoadjuvant RT or preoperative embolization, history of preoperative chemotherapy, spinal instability neoplastic score (SINS), Tomita score, and Tokuhashi score, were recorded. Furthermore, perioperative data, including the operation level, anterior column support, mean operation time, mean estimated blood loss (EBL), history of postoperative chemotherapy, history of adjuvant RT, radiological local control rate, complications, and follow-up period, were recorded. Furthermore, the results of questionnaire surveys, including the Spine Oncology Study Group Outcomes Questionnaire and visual analog scale, and doctor’s assessments, including Eastern Cooperative Oncology Group performance score, and Karnofsky Performance Score, were reviewed before surgery and at the last follow-up to evaluate health-related quality of life, degree of pain, and performance status.

Radiological local control was assessed by pre- and postoperative magnetic resonance imaging (MRI), by measuring the tumor volume on MRI. MRI was conducted before and 2 to 3 months after the surgery, and the response was classified as follows: decreased tumor (≥20% tumor volume reduction), stable disease (tumor volume reduction or enlargement <20%), or tumor progression (≥20% volume enlargement). Decreases in tumor volume or stable disease were indicative of tumor control. The overall survival (OS) was defined as the period from the date of surgery to the patient’s death due to disease or the last follow-up in August 2020. The tumor type was classified into 2 groups: favorable (i.e., prostate, breast, thyroid, and kidney) and unfavorable (i.e., lung, liver, colon, and pancreas), according to the known clinical profile of the tumor.^[13,14]^

Quantitative data were presented as the mean ± standard deviation unless otherwise indicated. Independent t-tests were used to assess the continuous variables, and chi-squared test or Fisher exact test were used to analyze the categorical variables. The Kaplan–Meier method was used to estimate the survival rate, with log-rank test used to identify the difference between surgical methods. A *P*-value >.05 was considered statistically significant. All statistical tests were 2 sided. *R* version 3.6.1 (*R* Foundation for Statistical Computing, Vienna, Austria) was used for all statistical analyses.

## 3. Results

### 3.1. Preoperative demographic data

A total of 105 patients were included in this study. The cohort consisted of 75 men and 30 women, with a mean age of 60.8 years (range: 25–81 years). The most frequent sites of primary tumor were the lung (19 patients; 18.1%), liver (16 patients; 15.2%), prostate (13 patients; 12.4%), and breast (10 patients; 9.5%). The thoracic level was the most common offending level of the tumor (53 patients; 50.5%) followed by cervicothoracic (22 patients; 21.0%) and lumbar level (18 patients; 17.1%). Neoadjuvant RT was delivered in 16 patients (15.3%) with either SRS (5 patients; 4.8%) or external beam radiotherapy (EBRT) (11 patients; 10.5%). Preoperative embolization was performed in 13 patients (12.4%), and preoperative chemotherapy was performed in 80 patients (76.2%). SINS score was also evaluated before surgical decision-making. Overall, 53 patients (50.5%) were classified as unstable (13–18 points), while 52 patients (49.5%) were classified as potentially unstable (7–12 points). Overall, 18 patients (17.1%) had a Tomita score of 2 to 3, 41 patients (39.1%) had a score of 4 to 5, 25 patients (23.8%) had a score of 6 to 7, and 21 (20.0%) had a score of 8 to 10. In addition, 64 patients (60.9%) had a Tokuhashi score of 0 to 8, 30 (28.6%) had a score of 9 to 11, and 11 (10.5%) had a score of 12 to 15 (Table 1).

**Table 1:** Preoperative clinical characteristics of the patients.

	TS	CPS	CTJF	TLSF	DS	Total
Number of patients	35	7	17	38	8	105
Age (SD), y	62.3 (12.4)	61.0 (13.1)	59.9 (12.4)	59.6 (13.4)	61.4 (10.2)	60.8 (12.5)
Sex (%)						
Male	22 (62.9)	4 (57.1)	11 (64.7)	32 (84.2)	6 (75.0)	75 (71.4)
Female	13 (37.1)	3 (42.9)	6 (35.3)	6 (15.8)	2 (25.0)	30 (28.6)
Site of primary tumor (%)						
Lung	10 (28.6)	0 (0.0)	5 (29.4)	4 (10.5)	0 (0.0)	19 (18.1)
Liver	4 (11.4)	4 (57.1)	0 (0.0)	8 (21.1)	0 (0.0)	16 (15.2)
Prostate	1 (2.9)	0 (0.0)	2 (11.8)	7 (18.4)	3 (37.5)	13 (12.4)
Breast	3 (8.6)	0 (0.0)	2 (11.8)	4 (10.5)	1 (12.5)	10 (9.5)
Kidney	6 (17.1)	0 (0.0)	2 (11.8)	1 (2.6)	0 (0.0)	9 (8.6)
Colon	4 (11.4)	0 (0.0)	1 (5.9)	2 (5.3)	0 (0.0)	7 (6.7)
Pancreas	1 (2.9)	0 (0.0)	2 (11.8)	1 (2.6)	0 (0.0)	4 (3.8)
Multiple myeloma	1 (2.9)	0 (0.0)	0 (0.0)	1 (2.6)	1 (12.5)	3 (2.9)
Bladder	0 (0.0)	0 (0.0)	1 (5.9)	2 (5.3)	0 (0.0)	3 (2.9)
Rectal	1 (2.9)	0 (0.0)	0 (0.0)	0 (0.0)	1 (12.5)	2 (1.9)
Sarcoma	0 (0.0)	1 (14.3)	0 (0.0)	1 (2.6)	0 (0.0)	2 (1.9)
Others	4 (11.4)	2 (28.6)	2 (11.8)	7 (18.4)	2 (25.0)	17 (16.2)
Offending level (%)						
Cervical	0 (0.0)	7 (100.0)	0 (0.0)	0 (0.0)	1 (12.5)	8 (7.6)
Cervicothoracic	4 (11.4)	0 (0.0)	17(100.0)	0 (0.0)	1 (12.5)	22 (21.0)
Thoracic	26 (74.3)	0 (0.0)	0 (0.0)	23 (60.5)	4 (50.0)	53 (50.5)
Thoracolumbar	2 (5.7)	0 (0.0)	0 (0.0)	1 (2.6)	1 (12.5)	4 (3.8)
Lumbar	3 (8.6)	0 (0.0)	0 (0.0)	14 (36.8)	1 (12.5)	18 (17.1)
Preoperative chemotherapy (%)	26 (74.3)	5 (71.4)	11 (64.7)	31 (81.6)	7 (87.5)	80 (76.2)
Neoadjuvant radiotherapy (%)						
Performed	7 (20.0)	3 (42.9)	1 (5.9)	5 (13.2)	0 (0.0)	16 (15.3)
SRS	1 (2.9)	2 (28.6)	1 (5.9)	1 (2.6)	0 (0.0)	5 (4.8)
EBRT	6 (17.1)	1 (14.3)	0 (0.0)	4 (10.5)	0 (0.0)	11 (10.5)
Not performed	28 (80.0)	4 (57.1)	16 (94.1)	33 (86.8)	8 (100.0)	89 (84.8)
Preoperative embolization	5 (14.3)	1 (14.3)	2 (11.8)	5 (13.2)	0 (0.0)	13 (12.4)
Spinal instability neoplastic score						
Score ≥13	21 (60.0)	5 (71.4)	14 (82.4)	13 (34.2)	0 (0.0)	53 (50.5)
7 ≤ score < 13	14 (40.0)	2 (28.6)	3 (17.6)	25 (65.8)	8 (100.0)	52 (49.5)
Tomita score						
2–3	3 (8.6)	0 (0.0)	4 (23.5)	6 (15.8)	5 (62.5)	18 (17.1)
4–5	10 (28.6)	5 (71.4)	6 (35.3)	18 (47.4)	2 (25.0)	41 (39.1)
6–7	12 (34.2)	1 (14.3)	2 (11.8)	9 (23.7)	1 (12.5)	25 (23.8)
8–10	10 (28.6)	1 (14.3)	5 (29.4)	5 (13.1)	0 (0.0)	21 (20.0)
Tokuhashi score						
0–8	20 (57.1)	6 (85.7)	11 (64.7)	22 (57.9)	5 (62.5)	64 (60.9)
9–11	10 (28.6)	1 (14.3)	4 (23.5)	12 (31.6)	3 (37.5)	30 (28.6)
12–15	5 (14.3)	0 (0.0)	2 (11.8)	4 (10.5)	0 (0.0)	11 (10.5)

CPS = cervical pedicle screw, CTJF = cervicothoracic junction fixation, DS = decompression surgery, EBRT = external beam radiotherapy, SD = standard deviation, SRS = stereotactic radiosurgery, TS = traditional surgery, TLSF = thoracolumbar short fixation.

### 3.2. Treatment

TS, CPS, CTJF, TLSF, and DS were performed in 35, 7, 17, 38, and 8 patients, respectively. The most common number of surgery levels was 2 (40 patients; 38.1%), followed by 4 (35 patients; 33.3%). Anterior column support was provided in 39 patients (37.2%), performed using either corpectomy with a mesh cage (11 patients; 10.5%), or discectomy with an interbody cage (28 patients; 26.7%). The mean operative time from skin opening to closure was 261.1 minutes (range 103 to 390 minutes). The mean EBL was 457.6 mL (range 50 to 900 mL) (Table 1). Postoperative chemotherapy was performed in 63 patients (60.0%). Postoperative adjuvant RT was considered in all patients and was performed in 53 patients (50.5%) with either SRS (21 patients; 20.0%) or EBRT (32 patients; 30.5%), who were regarded as eligible for adjuvant RT (Table 2).

**Table 2:** Perioperative clinical characteristics of the patients.

	TS	CPS	CTJF	TLSF	DS	Total
Number of patients	35	7	17	38	8	105
Number of surgery level (%) Mean	4.2	2.3	3.3	2.4	1.4	3.1
1	0 (0.0)	0 (0.0)	0 (0.0)	0 (0.0)	5 (62.5)	5 (4.8)
2	0 (0.0)	5 (71.4)	6 (35.3)	26 (68.4)	3 (37.5)	40 (38.1)
3	1 (2.9)	2 (28.6)	2 (11.8)	9 (23.7)	0 (0.0)	14 (13.3)
4	25 (71.4)	0 (0.0)	7 (41.2)	3 (7.9)	0 (0.0)	35 (33.3)
5	7 (20.0)	0 (0.0)	2 (11.8)	0 (0.0)	0 (0.0)	9 (8.6)
6	1 (2.9)	0 (0.0)	0 (0.0)	0 (0.0)	0 (0.0)	1 (1.0)
7	1 (2.9)	0 (0.0)	0 (0.0)	0 (0.0)	0 (0.0)	1 (1.0)
Anterior column support (%)						
Performed	22 (62.9)	0 (0.0)	7 (41.2)	10 (26.3)	0 (0.0)	39 (37.2)
Corpectomy with cage	9 (25.7)	0 (0.0)	2 (11.8)	0 (0.0)	0 (0.0)	11 (10.5)
Interbody cage	13 (37.1)	0 (0.0)	5 (29.4)	10 (26.3)	0 (0.0)	28 (26.7)
Not performed	13 (37.1)	7 (100.0)	10 (58.8)	28 (73.7)	8 (100.0)	66 (62.9)
Mean operation time (SD), min	305.8 (74.4)	264.1 (51.1)	286.6 (77.3)	221.7 (40.3)	195.5 (68.1)	261.1 (73.8)
Mean estimated blood loss (SD), mL	692.9 (552.6)	242.8 (127.2)	444.1 (321.6)	356.6 (351.8)	125.0 (84.5)	457.6 (441.8)
Postoperative chemotherapy (%)	18 (51.4)	3 (42.9)	12 (70.6)	27 (71.1)	3 (37.5)	63 (60.0)
Adjuvant radiotherapy (%)						
Performed	21 (60.0)	4 (57.2)	6 (35.3)	20 (52.6)	2 (25.0)	53 (50.5)
SRS	9 (25.7)	1 (14.3)	4 (23.5)	7 (18.4)	0 (0.0)	21 (20.0)
EBRT	12 (34.3)	3 (42.9)	2 (11.8)	13 (34.2)	2 (25.0)	32 (30.5)
Not performed	14 (40.0)	3 (42.9)	11 (64.7)	18 (47.4)	6 (75.0)	52 (49.5)

CPS = cervical pedicle screw, CTJF = cervicothoracic junction fixation, DS = decompression surgery, EBRT = external beam radiotherapy, SD = standard deviation, SRS = stereotactic radiosurgery, TS = traditional surgery, TLSF = thoracolumbar short fixation.

### 3.3. Postoperative outcomes

Of the 105 enrolled patients, 74 (70.5%) were available for a follow-up MRI, while 31 (29.5%) were unable to undergo follow-up MRI owing to death or loss to follow-up. The radiological response to surgery was classified as either decreased tumor, stable disease, or tumor progression, depending on the tumor volume between the preoperative and follow-up MRI. On MRI, decreased tumor volume was observed in 18 patients (17.1%), and stable disease in 42 patients (40.0%). These 2 groups (60 patients with 57.1%) were classified as having controlled tumors, comprising 81.1% (60 of 74 patients) of patients who were able to undergo follow-up MRI. These results indicate that the local tumor control rate was 81.1%. Another 14 patients (13.3%) showed tumor progression (Table 3).

**Table 3:** Postoperative clinical characteristics of the patients.

	TS	CPS	CTJF	TLSF	DS	Total
Number of patients	35	7	17	38	8	105
Radiological local control (%)						
Decreased tumor	9 (25.7)	1 (14.3)	2 (11.8)	4 (10.5) 21	2 (25.0)	18 (17.1)
Stable disease	12 (34.3)	4 (57.1)	4 (23.5)	(55.3)	1 (12.5)	42 (40.0)
Tumor progression	5 (14.3)	1 (14.3)	1 (5.9)	6 (15.8)	1 (12.5)	14 (13.3)
Follow-up loss	9 (25.7)	1 (14.3)	10 (58.8)	7 (18.4)	4 (50.0)	31 (29.5)
Complications (%)						
Total	1 (2.9)	1 (14.3)	1 (5.9)	3 (7.9)	0 (0.0)	6 (5.7)
Wound infection	1 (2.9)	0 (0.0)	0 (0.0)	0 (0.0)	0 (0.0)	1 (1.0)
Postoperative hematoma	0 (0.0)	0 (0.0)	0 (0.0)	2 (5.3)	0 (0.0)	2 (1.9)
Recurrence	0 (0.0)	1 (14.3)	1 (5.9)	1 (2.6)	0 (0.0)	3 (2.9)
Mean follow-up (SD), mo	7.9 (7.2)	4.3 (4.4)	7.4 (8.3)	7.2 (6.6)	7.0 (8.7)	7.3 (7.1)

CPS = cervical pedicle screw, CTJF = cervicothoracic junction fixation, DS = decompression surgery, SD = standard deviation, TS = traditional surgery, TLSF = thoracolumbar short fixation.

Surgical complications were documented in 6 patients (5.7%), with all patients requiring reoperation. One patient (1.0%) who received TS experienced wound infection and additional surgery was performed. Two patients (1.9%) who received TLSF showed postoperative epidural hematoma and required reoperation with no neurological deterioration. A further 3 patients (2.9%) showed tumor recurrence with spinal cord compression, which required additional surgeries (Table 3).

In addition, preoperative and postoperative comparisons of the results of questionnaire surveys (Spine Oncology Study Group Outcomes Questionnaire and visual analog scale) and doctor’s assessments (Eastern Cooperative Oncology Group performance score and Karnofsky Performance Score) showed a statistically significant difference (*P* < .001) (Table 4).

**Table 4:** Preoperative and postoperative comparisons of SOSGOQ score, VAS, ECOG-PS, and KPS.

	Preoperative score (SD)	Postoperative score (SD)	*P* value
SOSGOQ score	75.6 (8.3)	45.4 (8.4)	<.001
VAS	6.2 (1.8)	3.1 (1.5)	<.001
ECOG-PS	3.4 (0.5)	2.0 (1.0)	<.001
KPS	39.5 (11.5)	62.5 (18.3)	<.001

ECOG-PS = Eastern Cooperative Oncology Group performance score, KPS = Karnofsky Performance Score, SD = standard deviation, SOSGOQ = Spine Oncology Study Group Outcomes Questionnaire, VAS = visual analogue scale.

### 3.4. Survival analysis

**Figure 3.**
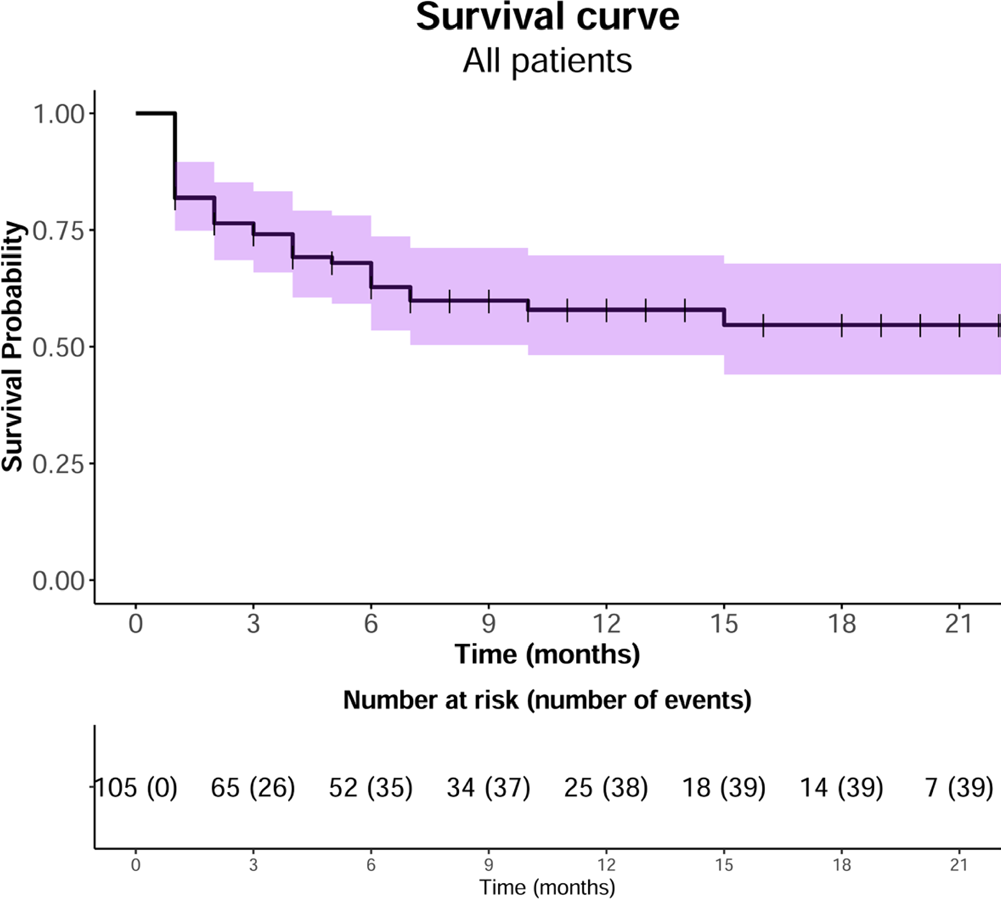
Kaplan–Meier survival curve showing a postoperative 1-year survival of 57.9%. The median overall survival is not reached at a mean follow-up of 7.3 mo.

**Figure 4.**
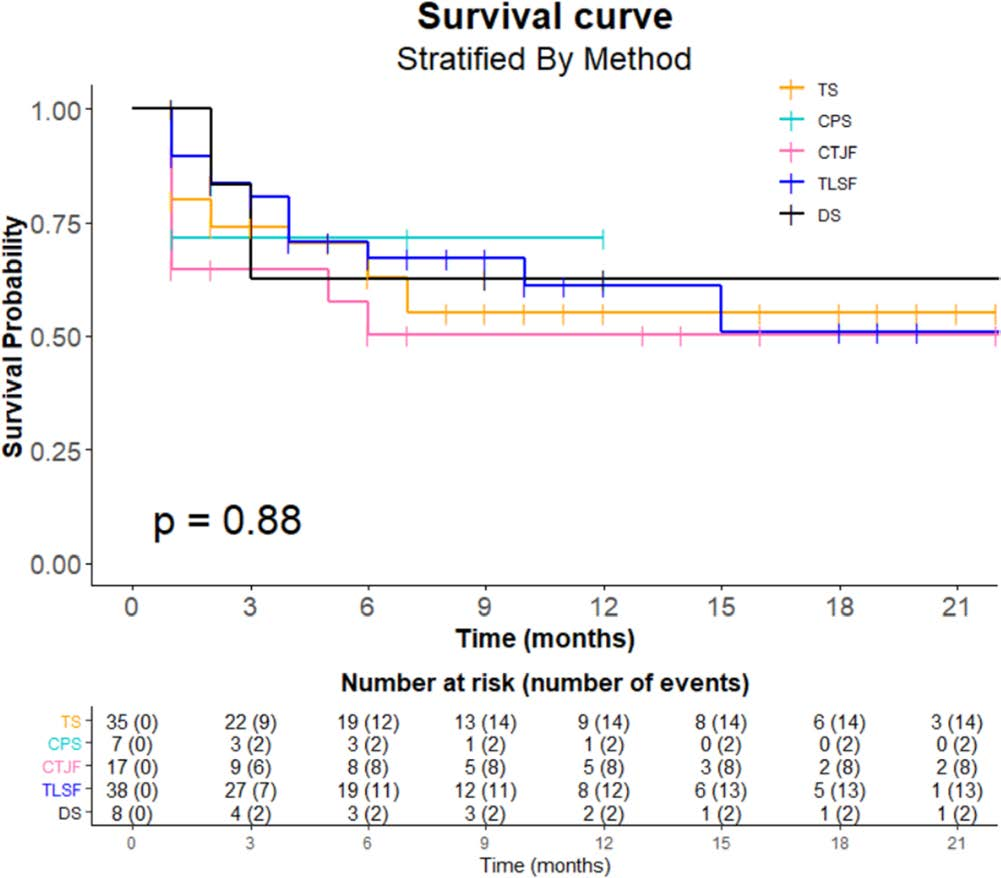
Kaplan–Meier survival curve stratified by the surgical methods shows no statistical difference between the methods with the log-rank test (*P* = .88).

**Figure 5.**
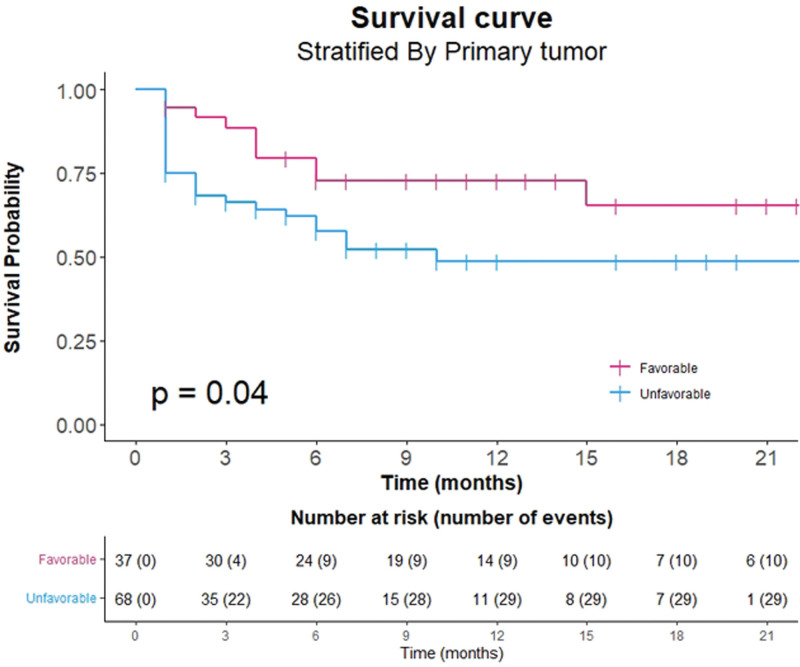
Kaplan–Meier survival curve stratified by the primary tumor shows a statistical difference between the primary tumor with the log-rank test (*P* = .04).

Of the 105 patients included in our study, 39 died, and 66 were censored (52 patients survived, and 14 patients were lost to follow-up). In the Kaplan–Meier survival curve, the OS rates were 74.1%, 62.8%, and 57.9% at 3, 6, and 12 months after surgery, respectively (Fig. 3). The median OS was not reached. The mean follow-up period was 7.3 months (range: 1–28 months) (Table 1). There was no statistical difference in the survival analysis between the surgical methods (*P* = .88) (Fig. 4). However, there was a statistically significant difference according to the type of the tumor in the survival analysis: the OS rates were 72.7% in favorable tumors and 48.6% in unfavorable tumors at 12 months after surgery (*P* = .04) (Fig. 5).

### 3.5. Representative scenarios

#### 3.5.1. Scenario 1.

**Figure 6.**
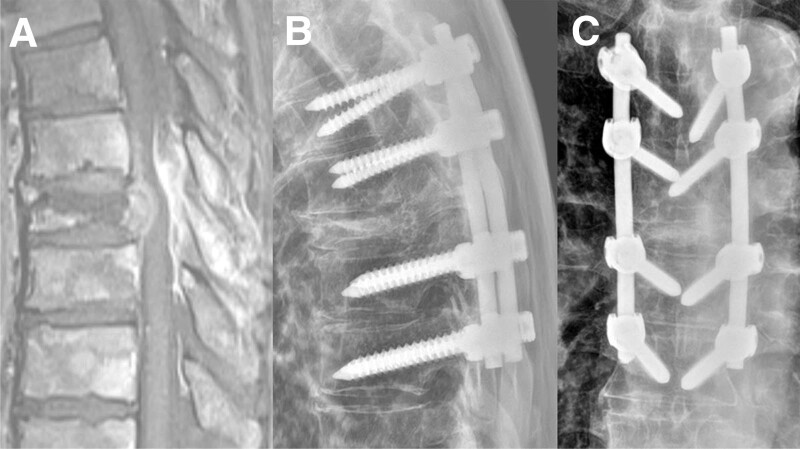
A 65-year-old man diagnosed with prostate cancer presented to the clinic with gait disturbance and back pain. Preoperative sagittal MRI (A) reveals T8 pathologic compression fracture and cord compression. TS is performed, and postoperative lateral (B) and AP (C) X-ray films show the posterior screw fixation from T6 to T10. AP = anteroposterior, MRI = magnetic resonance image.

A 65-year-old man diagnosed with prostate cancer presented to the clinic with gait disturbance and back pain. T8 pathologic compression fracture and cord compression was identified on MRI (Fig. 6A). Since the lesion was solitary and the general condition was good, TS involving removal of the cord compressive lesion and screw fixation from T6 to T10 was performed (Fig. 6B, C).

#### 3.5.2. Scenario 2.

**Figure 7.**
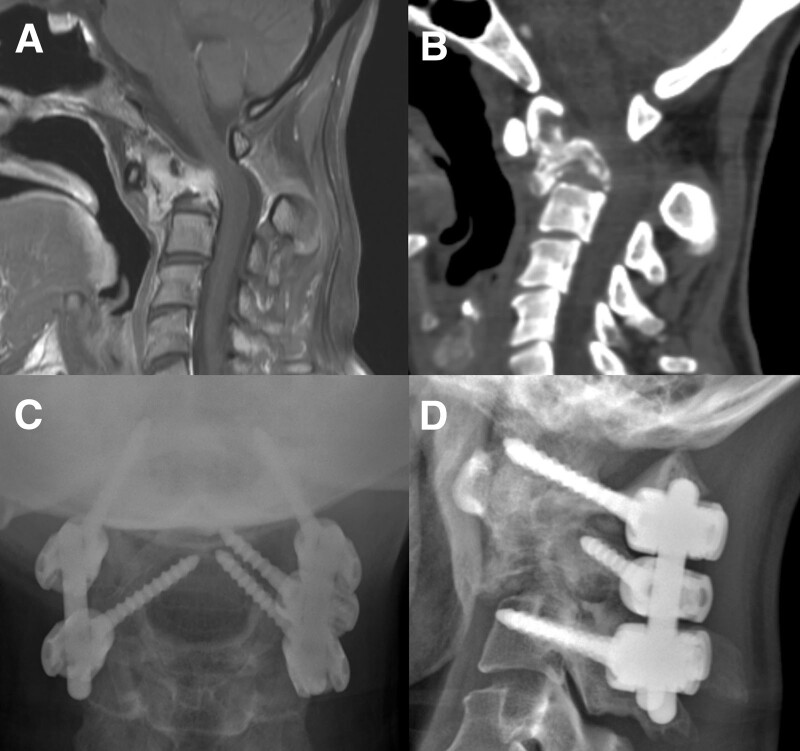
A 44-year-old woman diagnosed with breast cancer presented to the clinic with neck pain and a tingling sensation in both arms. Initial MRI (A) reveals a collapse of the C2 body with a metastatic tumor, and CT (B) shows an osteolytic tumor at the C2 body. Occiput-preserving CPS is performed from C1 to C3. Postoperative AP (C) and lateral (D) X-ray films show well-fixed pedicle screws. AP = anteroposterior, CPS = cervical pedicle screw, CT = computed tomography, MRI = magnetic resonance image.

A 44-year-old woman diagnosed with breast cancer presented to the clinic with neck pain and tingling sensation in both arms. Initial MRI revealed a collapse of the C2 body with a metastatic tumor, and computed tomography revealed an osteolytic tumor at the C2 body (Fig. 7A, B). Occiput-preserving CPS was performed from C1 to C3 (Fig. 7C,D). Her pain and tingling sensation improved after surgery without any complications.

#### 3.5.3. Scenario 3.

**Figure 8.**
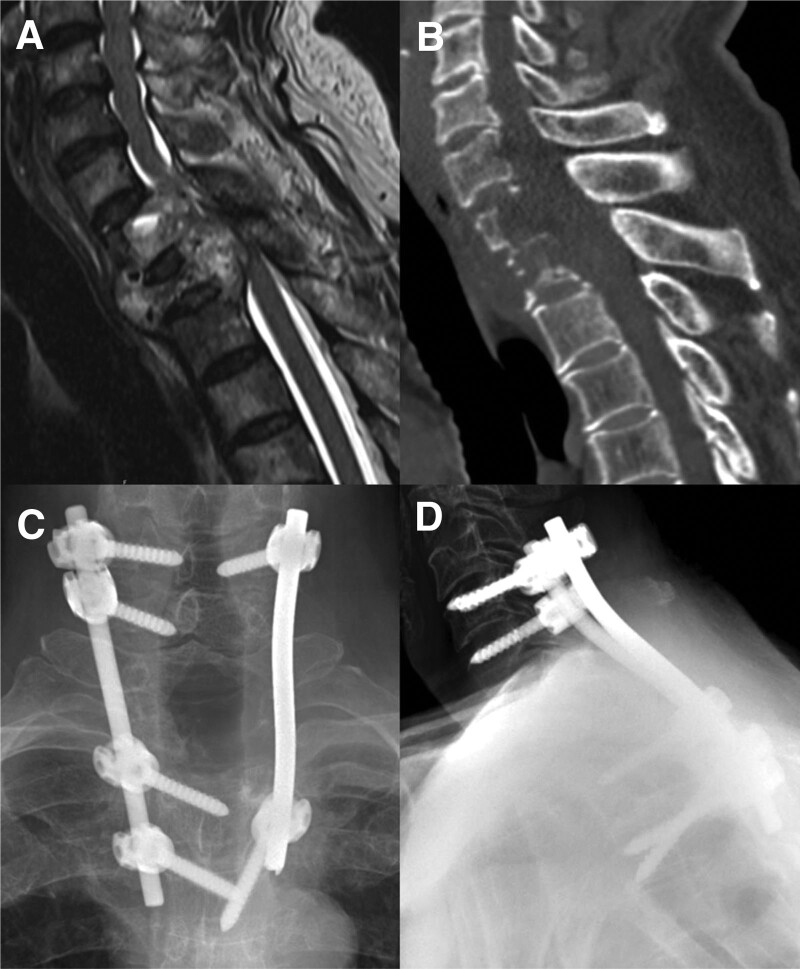
A 64-year-old man diagnosed with kidney cancer presented to the hospital with gait disturbance and severe back pain. Preoperative sagittal MRI (A) and sagittal CT (B) reveals the osteolytic metastatic spinal tumor at T1–2, compromising the spinal cord. Tumor removal with CTJF using 5.5-mm-diameter rods is performed from C6 to T4. Postoperative anteroposterior (C) and lateral (D) X-ray films show well-fixed pedicle screws at the cervicothoracic junction. CT = computed tomography, CTJF = cervicothoracic junction fixation, MRI = magnetic resonance image.

A 64-year-old man diagnosed with kidney cancer came to the hospital with gait disturbance and severe back pain. MRI and computed tomography revealed an osteolytic metastatic spinal tumor at T1–2, which was compromising the spinal cord (Fig. 8A, B). Posterior instrumentation with tumor removal was performed. As the procedure is performed in the cervicothoracic junction, 5.5-mm-thick-diameter rods were used to allow strong fixation from C6 to T4 (Fig. 8C, D). At follow-up, the patient’s gait disturbance had improved, and the pain had also subsided.

#### 3.5.4. Scenario 4.

**Figure 9.**
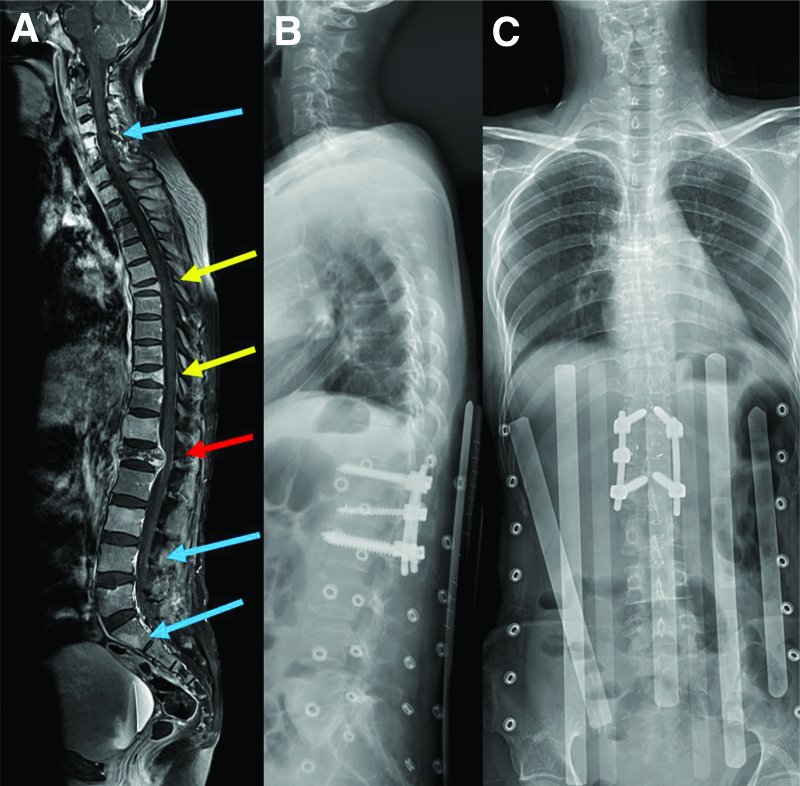
A 37-year-old woman diagnosed with parotid gland cancer presented to the hospital with sudden leg weakness and severe back pain. Preoperative sagittal MRI (A) reveals several vertebral metastases, including at the C4–6, L4, and S1 vertebral body masses (indicated by blue arrows); T6 and T10 pathologic compression fractures (indicated by yellow arrows); and L1 pathologic compression fracture (indicated by a red arrow). The presence of adjacent metastatic lesions made it difficult to perform the traditional long level surgery, therefore, we performed TLSF at the offending L1 level. Postoperative lateral (B) and anteroposterior (C) X-ray films show well-fixed screws at T12 to L2. MRI = magnetic resonance image, TLSF = thoracolumbar short fixation.

A 37-year-old woman diagnosed with parotid gland cancer presented to the hospital with sudden leg weakness and severe back pain. She had already been diagnosed with metastases in different vertebral regions, including C4–6, L4, and S1 vertebral body masses, and T6, T10, and L1 pathologic compression fractures. MRI revealed a collapsed L1 body with spinal cord compression (Fig. 9A). The presence of adjacent metastatic lesions made it difficult to perform traditional long level surgery; therefore, we performed the TLSF at the offending L1 level. The patient underwent a maximal debulking surgery with TLSF at T12 to L2 (Fig. 9B, C). After the operation, the motor weakness improved, and pain was controlled by medication.

#### 3.5.5. Scenario 5.

**Figure 10.**
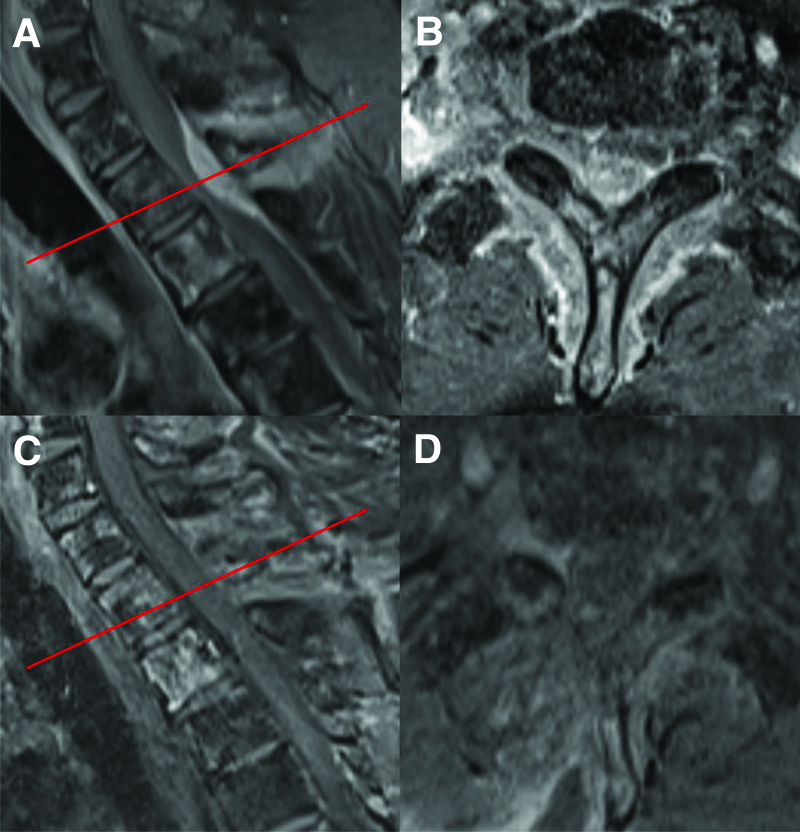
A 59-year-old man diagnosed with prostate cancer presented to the clinic with gait disturbance and pain and tingling sensation in both arms. Preoperative sagittal (A) and axial (B) MRI show a metastatic epidural mass, which compresses the spinal cord at the T1–2 level. As the facet joint and pedicles are intact, DS without fixation is performed. Follow-up sagittal (C) and axial (D) MRI show total removal of the tumor. Each sagittal level of the axial images is indicated by red lines. DS = decompression surgery, MRI = magnetic resonance image.

A 59-year-old man diagnosed with prostate cancer presented to the clinic with gait disturbance and pain and tingling sensation in both arms. MRI revealed a metastatic epidural mass compressing the spinal cord at the T1–2 level (Fig. 10A, B). DS without fixation was conducted, as the facet joint and pedicles were intact with posterior column compression only. Follow-up MRI revealed total removal of the tumor (Fig. 10C, D), and the patient’s gait disturbance, arm pain, and tingling sensation were improved.

## 4. Discussion

Recent advances in cancer treatment have allowed increased survival time for patients, resulting in more patients developing spinal metastasis.^[3]^ Although other treatment modalities (e.g., RT, SRS, and chemotherapy) have undergone significant development, surgery remains the most important method in treating the metastasis, especially in urgent situations. As surgical techniques and devices develop, surgical treatment has been widely performed in patients with spinal metastasis for restoration of neurological deficits, stabilization of the spinal column, and pain management. Traditionally, spinal metastasis has been treated using posterior fixation at 2 levels above and 2 levels below the affected vertebrae. However, because of the clinical, anatomical problems, and burden to the patients, it is sometimes difficult to perform TS. Moreover, sometimes this can induce undesirable complications, especially in patients with a poor general condition.^[6,7]^

It is well known that survival of the metastatic spinal tumor is dependent on the primary tumor.^[13,15]^ We also found that the survival rate is affected by the clinical characteristics of the primary tumor rather than by the surgical methods. Although the surgical method does not entirely affect survival, it plays an essential role in controlling the tumor efficiently. In metastatic tumors, surgery is performed to alleviate the symptoms and restore the patient’s functional abilities.^[16]^ This principle is becoming increasingly important as the survival time of patients is increasing. The main purpose of the surgical treatment should be to achieve decompression of the neural structure from the tumor, which allows maximal tumor reduction, rather than total resection.^[17,18]^ In addition, whether fusion is necessary in the treatment of metastatic spinal tumor is still under debate, as current survival rates are insufficient for instability problems to occur.^[11,12]^

In our study, various surgical methods were used to manage spinal metastases, depending on the characteristics of the tumor, considering the amount of tumor invasion and life expectancy of the patients. TS was performed in patients who had single solitary metastasis with a clinically good performance status. CPS with preservation of the occiput was administered in patients with cervical metastasis. This makes the extent of the surgery smaller and less aggressive. CTJF was performed in the cervicothoracic junction using firm instrumentation. In the cervical spine firm instrumentation was secured even in surgically challenging situations owing to the technical achievement of the pedicle screw insertion.^[19–22]^ TLSF was performed in patients with multiple spinal metastasis at the adjacent level. Since it is challenging to determine the surgical level when another metastatic lesion also exists in the adjacent vertebra, short fixation instead of TS was performed in the thoracolumbar spine. DS was performed when the structures related to stability, including the facet joints and pedicles, were well preserved without invasion of the metastasis.

The Tokuhashi score has been most widely used to predict the survival of patients with a metastatic spinal tumor. We did not, however, rely solely on this scoring system. We decided on surgical treatment by considering the variable statuses of the patients. This is because some patients with a Tokuhashi score of <8 have an opportunity to recover owing to recent advances in systemic treatment.^[23]^ Therefore, the Tokuhashi score could not be used as a definitive scale for the management of spinal metastasis.

Deciding the tumor resection margin is difficult in spinal metastasis surgery. The Tomita score and Tokuhashi score, which are well-known scoring systems to evaluate the general status of patients with a metastatic spinal tumor, were considered when deciding which treatment should be performed.^[24–26]^ Although the surgical margin was decided based on these scoring systems (i.e., the lower the Tomita score and the higher the Tokuhashi score, the more radical surgery was performed), we also considered the characteristics of the tumor. Since bone metastasis is classified as osteolytic, osteoblastic, or mixed, depending on the basic feature of bone remodeling,^[1]^ osteolytic tumors which can be easily removed using conventional surgical instruments are removed intraoperatively. However, the osteoblastic bone, which is sufficiently solid to maintain the vertebral body, was not removed, and was instead used as a supportive structure for screw insertion. In addition, to achieve solid stability after the tumor resection, anterior reconstruction is sometimes needed in the case of the vertebral body or when pedicles are disrupted because of the tumor invasion. This situation was more obvious in the case of osteolytic tumors than in osteoblastic tumors as the former are less stable, especially after decompression. Therefore, considering the general status of the patients and characteristics of the tumor, we decided on the degree of tumor resection using the following procedures: total en bloc spondylectomy, intralesional spondylectomy, piecemeal spondylectomy, or DS. Since the available scoring systems cannot clarify all details of the tumor, we also decided on optimal clinical decisions for resection of the tumor with consideration of the intraoperative tumor characteristics.

In our study population, 76% of the patients underwent preoperative chemotherapy, and 60% underwent postoperative chemotherapy. As oncological treatment develops over time, more patients are receiving chemotherapy for primary tumor control. However, if the primary tumor is resistant to chemotherapy and the general status of the patient is not sufficient to overcome the chemotherapy, chemotherapy was not chosen for the treatment. In addition, the patients who were first diagnosed with a primary tumor as a metastatic spinal tumor that needs emergent surgery could not have the chance for preoperative chemotherapy. Overall, chemotherapy was usually considered and performed for primary tumor control. Postoperative chemotherapy was selected less commonly than preoperative chemotherapy, as many patients were in the later stages of disease with poor general condition when diagnosed with spinal metastasis.

There were several limitations in this study. First, the primary tumors of the patients with spinal metastasis were heterogenous. These differences in primary tumor would have influenced the prognosis and outcome of patients. Furthermore, other treatments apart from surgery, also differed between patients. Second, because this study is a retrospective observational study, randomization of each surgical method could not be performed. Third, only patients who underwent a surgical intervention as treatment for spinal metastasis were included in the study. Hence, caution should be observed when generalizing the prognosis, especially in patients with spinal metastasis who cannot undergo surgery.

## 5. Conclusion

We performed various surgical methods to treat metastatic spinal tumors and observed good results. Our results indicate that it is not inferior to apply surgical methods other than TS in managing spinal metastasis. It is important to perform an individualized and interdisciplinary decision-making when treating spinal metastasis.

## Author Contributions

Conzeptualization: Jin Hoon Park.

Data curation: Hong Kyung Shin, Myeongjong Kim, Subum Lee, Jung Jae Lee, Danbi Park.

Formal analysis: Hong Kyung Shin, Myeongjong Kim.

Investigation: Myeongjong Kim, Subum Lee, Jung Jae Lee.

Methodology: Hong Kyung Shin, Myeongjong Kim.

Software: Myeongjong Kim, Danbi Park.

Writing – original draft: Hong Kyung Shin, Myeongjong Kim.

Writing – review & editing: Hong Kyung Shin, Sang Ryong Jeon, Sung Woo Roh, Jin Hoon Park.
